# Development of an online information and support resource for adolescent idiopathic scoliosis patients considering surgery: perspectives of health care providers

**DOI:** 10.1186/1748-7161-5-13

**Published:** 2010-06-29

**Authors:** Radha MacCulloch, Joyce Nyhof-Young, David Nicholas, Sandra Donaldson, James G Wright

**Affiliations:** 1 The Hospital for Sick Children, Toronto, Ontario, Canada; 2 Princess Margaret Hospital, Toronto, Ontario, Canada; 3 University of Calgary, Faculty of Social Work, Edmonton, Alberta, Canada

## Abstract

**Background:**

Adolescents with idiopathic scoliosis who are considering spinal surgery face a major decision that requires access to in-depth information and support. Unfortunately, most online resources provide incomplete and inconsistent information and minimal social support. The aim of this study was to develop an online information and support resource for adolescent idiopathic scoliosis (AIS) patients considering spinal surgery. Prior to website development, a user-based needs assessment was conducted. The needs assessment involved a total of six focus groups with three stakeholder groups: (1) post-operative AIS patients or surgical candidates (10-18 years) (n = 11), (2) their parents (n = 6) and (3) health care providers (n = 11). This paper reports on the findings from focus groups with health care providers.

**Methods:**

Focus group methodology was used to invite a range of perspectives and stimulate discussion. During audio-recorded focus groups, an emergent table of website content was presented to participants for assessment of relevance, viability and comprehensiveness in targeting global domains of need. Specifically, effective presentation of content, desired aspects of information and support, and discussions about the value of peer support and the role of health professionals were addressed. Focus group transcripts were then subject to content analysis through a constant comparative review and analysis.

**Results:**

Two focus groups were held with health care providers, consisting of 5 and 6 members respectively. Clinicians provided their perceptions of the information and support needs of surgical patients and their families and how this information and support should be delivered using internet technology. Health care providers proposed four key suggestions to consider in the development of this online resource: (1) create the website with the target audience in mind; (2) clearly state the purpose of the website and organize website content to support the user; (3) offer a professionally-moderated interactive support component; and (4) ensure accessibility of website information and support by considering the age, gender, reading level and geographic location of potential users.

**Conclusions:**

Health care providers collectively identified the need for the development of an online information and support resource for adolescents considering surgery for AIS and their families and described the proposed website as a positive and needed adjunct to current clinical care.

## Background

Adolescent idiopathic scoliosis (AIS) is a structural lateral curvature of the spine arising during puberty in otherwise normal children [1]. While epidemiologic studies estimate 1-3% of adolescents (10 to 18 years) will develop scoliosis, only 1-5% of these adolescents will receive spinal instrumentation and fusion for progressive curvature of more than 40°-50°. The clinical course of untreated scoliosis as an adult is controversial [1]. However, significant curves can cause deformity, increased frequency (although not necessarily severity) of back pain, decreased objective pulmonary function tests and in some patients, respiratory symptoms [2]. Surgery involves placing rods, straightening, and fusing the spine. In addition to the loss of spinal motion, the surgery has potential complications of infection, nonunion, back pain, and paraplegia [1]. The decision about surgery should involve consideration of the potential risks of both surgery and the clinical course of untreated scoliosis. Accordingly, considering spinal surgery for AIS involves a major decision for patients and their families, one that requires access to in-depth information and support throughout the decision-making process, surgery itself and recovery [3].

As Leonard and colleagues suggest, patients generally expect two things from the health care system: care and information [4]. While most care must occur in-person, information regarding health care, including both general and personal health information and support in decision-making and illness management may be delivered in-person and/or via hard-copy or online resources. The internet has dramatically transformed how we deliver and share information. The internet allows information to be easily and widely accessed, updated, and shared. In health care, it can connect health care providers, health care institutions and patients. It may further serve to connect patients with one another, an exceptional capability, particularly for those who may be isolated due to illness severity, an uncommon diagnosis or geography. Therefore, it is not surprising that web-based resources are increasingly part of Canadians' experience of health care. In 2005, approximately 8.7 million Canadian adults went online to search for medical or health related information [5]. Increasing use of the internet specifically for orthopaedic patients is also noted [6-8]. For instance, use of the internet for health information was reported by 55% of outpatients receiving an orthopaedic consultation. Of these, 52% had obtained online medical information, and 36% stated that they would accept medical consultation online [6]. Health care institutions offering orthopaedic services and individuals with scoliosis have also used the internet to share and access information and support regarding scoliosis. These forums/media range from websites sharing professionally-delivered information regarding scoliosis and treatment to personal blogs chronicling an individual's unique experience living with scoliosis to networking sites such as Facebook which hosts a group called, "How to Look Good Twisted". These all serve to share information regarding scoliosis and connect orthopaedic health care providers and individuals with scoliosis. Furthermore, preliminary research demonstrates that the delivery of health care information via the internet has been linked to positive patient outcomes, user satisfaction and cost-effectiveness [9,10].

As an open-sourced, often un-moderated and evolving medium, the internet has a seemingly limitless and expanding capacity to share information and connect people. However, these very benefits may also pose significant challenges. Despite extensive online utilization for health purposes, accuracy of information is often suspect [11-14]. A quick internet search on any health topic often reveals a staggering quantity of information of varying degrees of credibility. This is particularly concerning given that presentation and graphic quality influence use more than the integrity of website content [15-19]. Website quality and mode of presentation are therefore critical if websites are to be used effectively. Unfortunately, most current online resources emulate the deficiencies of current clinical practice by providing incomplete and inconsistent information and minimal or no venue for social support. For a patient and their family, this can lead to frustration and misinformation, which in turn may hinder informed decision-making.

A review of traditional (non-web-based) patient information and support literature in scoliosis using conventional retrieval sources such as MedLine, CINAHL, M(edica) (European and Asian Orthopaedics and Rehabilitation search engine), as well as web-based resources using health website search engines (e.g., sympatico http://www1.sympatico.ca, Yahoo Health http://www.yahoo.com/Health/Children_s_Health) was conducted in 2005 to determine the quantity and quality of existing information and support resources for AIS patients and their families. This review comprised a quantitative competitive analysis using the DISCERN, a 16-item standardized instrument [20,21] that assesses the quality of written information about treatment and health websites. A total of 22 websites were included in the review. Each website was independently evaluated by 2 reviewers. Only 7 out of the 22 websites scored at least 70% or higher on the DISCERN with a mean overall score of 63/80. However, the quality of these 7 websites remained compromised by unclear and/or unmet aims, lack of relevance to consumers, currency of information and unclear and unbiased sources of information. Specifically, this analysis revealed existing websites tend to lack a source (i.e. a reference), content based on levels of evidence, and specific Canadian content. Websites demonstrated failure to identify content authorship, poor graphics, poor readability and appeared to target only females. Several websites integrated product endorsement, thereby introducing a potentially confusing and biased mix of patient/parent information and industry promotion and benefit. None of these websites appeared to have formally considered the needs of families [22]. Furthermore, none of the existing scoliosis websites provided any specific family support beyond text information. Thus, this competitive analysis revealed an overall lack of evidence-based information, poor usability, and no mechanisms for family support. In addition, as expected with primarily U.S. based websites, the information provided was not particularly relevant to the Canadian Health Care context. This review also revealed a lack of useable, interactive online opportunities that (1) catered to both patients and parents, (2) integrated an array of evidence-based information and support, (3) was interactive and engaging, and (4) provided health professional moderated peer-based support. This review revealed a clear need for a credible, regionally-relevant information and support resource tailored to both male and female patients considering surgery for scoliosis and their families.

In redressing the gaps currently existing in online information and support for adolescents with AIS, the purpose of this study was to develop an online information and support resource for patients and their families considering AIS surgery. Because of limited face-to-face clinic time and the importance of informed decision-making, finding comprehensive yet efficient ways to provide the patient and their family with information and support should be considered a priority. Furthermore, the development of such a website has the potential to increase knowledge, social support and coping for patients considering surgery for AIS and their families. It further offers the potential to serve as a learning tool for health professionals by offering scoliosis-based educational content, providing access to appropriate links, and exemplifying an innovative patient-centred model for knowledge transfer. Finally, the website may move beyond simple information provision by adding an integrated mechanism for professionally-moderated online peer support.

## Methods

As part of a larger multi-phased project aimed at designing and evaluating an evidence-based online network for adolescents with AIS, this manuscript presents findings from a user-based needs assessment comprising health care provider focus groups. Specifically, initial focus groups previously had been conducted with adolescents with AIS and their parents [23]. In this paper, we are solely examining the perspectives of orthopaedic health care providers, elicited in focus groups, in which participants were invited to describe: (i) information and support needs of adolescents when considering surgery for scoliosis, and (ii) how this information and support can be optimally delivered using internet technology. Findings ultimately have been used in furthering design and subsequent evaluation plans for an evidence-informed information and support website for adolescents affected by AIS, and their families.

In this phase of this larger project, two focus groups of 6-8 inter-professional health care providers per group were conducted. Focus group size was established based on the guidelines outlined by Merton, Fiske and Kendall [24,25]. The focus groups each lasted approximately 90 minutes and were facilitated by two experienced focus group facilitators (both present in each group). Participants provided an extensive array of perspectives, and saturation of the data was reached. Informed consent was obtained from all participants prior to study participation, and institutional research ethics approval was received prior to study commencement.

### Focus Group Content

Focus groups were guided by a focus group schedule outlining the following open ended questions and probes. Accordingly, this schedule offered topical focus, but participants also had sufficient latitude to identify and address issues of salient interest, relevance, preference and priority. Specific focus group questions, based on this focus group schedule, comprised the following:

What are the needs of the patients/families?

•What do children with scoliosis need to help them with this condition?

•What do parents of children with scoliosis need to help them with this condition?

•What do others in the family need to help them with this condition and its impact on the family?

Have you used the internet to find scoliosis websites?

•How could online or web-based sources be helpful to families?

•What would need to be in a website for it to be most helpful to patients/families?

•What would prompt families to use the computer for information or support?

•What are the limitations to accessing a computer?

What would be an ideal website?

•If you were to design a table of contents for a website, what would you include?

•What do you think of the topics/information presented in this table of contents? [Potential table of contents presented]

•For each identified area, please indicate if you believe this idea is relevant, important, and clear.

•Please comment on the ordering of areas in the table of contents. What changes should be made?

During the focus groups, a preliminary table of contents, informed by clinical experience and a critical analysis of existing websites and relevant literature, was presented to participants for their assessment of its relevance, viability and comprehensiveness in targeting global domains of need. Specifically, effective presentation of content, desired aspects of information and support, and discussions about the value and format of peer support and the role of health professionals in internet-based education were initiated.

### Data Analysis

Focus groups were audio-recorded and transcribed to record participant feedback in as much detail as possible and to allow the facilitator to focus their attention on the group [26]. A research assistant documented key issues of group process (e.g., intra-group agreement/disagreement) in extensive field notes compiled during group sessions. Focus group transcripts were subjected to established methods of content analysis and interpretation [26,27]. Emerging patterns and concepts, as well as inconsistent or unclear data, were noted. A set of preliminary codes for text segments was then developed. After preliminary codes were applied to the data, a coding frame was developed to identify thematic patterns and conceptual linkages [27]. Inter-rater reliability was achieved between two experienced qualitative researchers who independently coded the same transcripts until a consistent understanding and application of codes was achieved. The development of the coding frame and themes, as well as the assessment of theoretical meaning and conceptual linkages, involved both analysts and the principal investigator. Discrepancies were specifically noted, discussed and resolved through consensus, and coding proceeded in a systematic and iterative process in which the coding frame served as an organizing resource; however, the team remained open to emergent findings through data review.

Finally, trustworthiness of study findings was sought. Fundamentally, the aim of establishing trustworthiness in qualitative research is to conduct research such that "*the findings of an inquiry are worth paying attention to*" (p. 290) [28]. In this study, trustworthiness of findings was demonstrated through: (i) coding clarification and consensus as outlined above, (ii) interpretation verification through the inclusion of text quotes, (iii) member checking whereby study findings were reviewed by a sub-sample of study participants and, (iv) peer debriefing among research team members and suitable clinicians, educators and technology consultants [28,29].

## Results

Focus group participants comprised interprofessional health care providers; one group with 5 participants and the other with 6 participants. Participants included three registered nurses, two physiotherapists, two orthotists, an orthopaedic surgeon, nurse practitioner, social worker, and child life specialist. Participants represented a range of both in and outpatient experience in orthopaedics and held between 1 and 20 years of paediatric orthopaedic experience (mean 8.4 years).

Health care providers identified a strong need for the development of an online information and support resource for adolescents considering surgery for AIS. They anticipated that a comprehensive and credible online resource to which they could refer patients and families could offset misinformation and/or confusion that patients and their families might encounter through other internet searches. One clinician explained, "*Lots of people come in to clinic and have 'Googled'. The concern is, are they appropriate sites or the appropriate articles? So this way [with the development of a new site], you're saying [to patients and their families], this is what we think is appropriate*." Furthermore, the current clinical practice of most health care institutions to address informational needs includes: appointments with physicians, teaching sessions with nurses and other healthcare providers, and informational resources such as pamphlets. Clinical appointments, however, are relatively brief, and often follow exceedingly long waits in the clinic and radiology, leaving little time for full and complete discussions. Health care providers noted that having an online resource would allow patients and their families time to digest the information received in clinic and to review the information again later online at a time and location that was convenient for them.

Health care providers identified the importance of customizing the content and design of the website to the identified users to maximize its utility for adolescents, families and health care providers alike. Health care providers also identified the following issues as the most pertinent to the adolescent surgical candidate and their families: information about the decision-making process, the surgery itself, post-operative medical procedures and the recovery period. Discussion centred on several key concerns which health care providers felt were most important to the development of an outstanding online information and support resource for adolescents considering surgery for AIS and their parents:

○ Creating the website with the target audience in mind

○ Home page & site navigation

○ Content: Information

■Decision-making and surgery

■Surgery and post-operative procedures

■Recovery

○ Content: Support

■Means of Support

■Sources of Support

○ Graphic design

○ Accessibility

### Creating the website with the target audience in mind

Health care providers emphasized the importance of user-centred design and considering the target audience throughout all phases of the development of the online information and support resource. From a clinical perspective, tailoring the website's content, format, style and page layout to the tastes and needs of the target population was identified by health care providers to be fundamental to supporting patients and their families throughout the decision-making process, surgery and recovery. Health care providers from both focus groups emphasized that the website should be clearly and explicitly aimed at adolescents with idiopathic scoliosis who are *considering *surgery as treatment and their families.

### Home page & site navigation

The careful creation of a home page and site navigation tools was identified as a necessary component of website development. Health care providers suggested that an introductory website homepage should inform users of the purpose and focus of the website and describe and discuss the criteria necessary to be considered a surgical candidate. One clinician proposed, *"I think you do need to have some criteria for who we're talking about [clearly posted on the website]. So when somebody clicks into the website [they can immediately determine], 'Do I fit these parameters?'"*. In addition, clinicians stressed that website content be organized differentially according to user (adolescent or parent) so that the information of greatest interest to the specific users should be easily identifiable and accessible. Health care providers identified parents as being primarily interested in learning about scoliosis and possible long-term surgical outcomes, while teens were concerned about how scoliosis and possible surgery could impact their lives in the short-term. It was suggested that website information should be organized accordingly, using a site map on the home page to 'filter' information according to the user and their needs. Imagining the experiences of an adolescent, one health care provider stated,

"If I was [sic] a teenager and I was thinking about surgery, I don't care about most of this stuff. My parents will figure it out. I want to go to that one bullet that says, 'How is this going to affect my life?', 'When do I get back to being with my friends?', 'When do I get back to my sports?', 'What am I going to look like at the end of it?'"

Another health care provider suggested organizing *"the knowledge systematically for people...Your teenager is not going to come in and say 'Hmmm...the history of scoliosis...interesting! Classification! Long Latin words that don't apply to me! Hmmmm...this is a really good website!' [Instead] they want to see the inside of someone's back, they want to know what's happening to them."*

### Content: Information

The informational content of the website was discussed in great detail by health care providers. They identified that informational content should be structured hierarchically, in order of importance to the person accessing the site. Based on their clinical experience, they reported that adolescent surgical candidates should be provided with in-depth educational information about surgery, with less of a focus on general information regarding scoliosis. Specifically, areas identified by health care providers as being of central importance to these teens included: decision-making regarding surgery, surgery and post-operative medical procedures, and recovery.

#### Decision-Making and Surgery

Health care providers stressed the importance of equipping the patient and/or parents with information necessary to make an informed decision regarding surgery. Specifically, listing general criteria to be considered by a candidate for surgery, potentially beneficial *and *challenging outcomes of surgery, as well as testimonials from adolescents about how they ultimately decided to proceed with surgery were recommended.

"There could be an audio [component], where one teen tells about their experience and how they made one decision versus another, because I think teens would like to hear their peers tell them things, as opposed to [adults]"

Based on clinical experience, one health care provider explained what a teen considering surgery for AIS might want to read or look at online: *"It's like, 'Show me the operation...What's going to happen to me?...What's good about this? What's bad about this? What do I need to do next?"*

While health care providers emphasized the value of listing potential benefits and risks of surgery on the website, they also stressed that the decision to undergo surgery should be considered a highly personal and individual process, as outcomes and perceived benefits and risks vary.

"*The [adolescents] that I've seen that have been most upset and the most surprised have been the ones who were synchronized swimmers or gymnasts who required a lot of [flexibility,] and then they came out and didn't realize that they couldn't do that anymore."*

#### Surgery and Post-Operative Procedures

Health care providers felt that the website should provide thorough descriptions of specific surgical and post-operative procedures, as well as introduce the potential surgical candidate to the health care team. Information on instrumentation, staged surgeries, post-operative traction and bracing and adjunctive treatments should be clearly described. Health care providers felt that the website might serve to further prepare teens by introducing them to the inter-professional health care providers that they may meet during their hospital stay and post-operative clinic visits. It was noted by participants that this section should be designed so as to be pertinent to potential surgical candidates across Canada; therefore varying instrumentation techniques and health care professions should be represented. For example, one clinician noted the importance of including information about adjunctive treatment providers such as "*the guy that gives you vitamins or chiropractors, or the people that sell you shoes, or the massage therapist*."

Furthermore, another health care provider suggested including information regarding potential patient involvement in research such as clinical trials and how participation in research may interact with clinical care.

#### Recovery

Post-surgical recovery in-hospital and at home were identified by health care providers as key topics for inclusion on the site. Information regarding the in-hospital recovery process should cover topics such as post-operative pain, the possible range of length of hospital stays and general hospital-related procedures and expectations for the post-operative course. Information regarding the experience of recovering at home should address 'getting back to normal' and the impact surgery may have on school and extra-curricular activities, including sports and time spent with friends (post-surgery appearance, coping and possible post-operative complications). Health care providers stressed that teens may feel uncomfortable bringing up questions related to the resumption of activities such as smoking or sexual activity. In these cases, health care staff suggested that these questions could be addressed on the site in a Frequently Asked Questions section and through links to other specialized websites.

Post-surgery appearance should be addressed in depth through inclusion of before and after photos or medical drawings of adolescents with idiopathic scoliosis that demonstrate the change in spine curvature and the length and visibility of the surgical scar. Representation of potential variations in surgical outcomes should be included.

"Number 1 is, what am I going to look like? How long is the scar going to be there? Will it be visible through my clothes? Once I go back to school what sort of activities can I do? How big is the scar going to be? When can I go back to school? Can I go out into the sun, swimming?"

Health care providers discussed their observations of friction or conflict between teen and parent(s) in post-operative clinic visits as evidence of the significant shifts that can occur within a family following surgery for scoliosis. Specifically, roles and boundaries within the family dyad may shift with the adolescent's increased dependence on parents and parent's increased protectiveness or attention. These changes may be particularly evident immediately post-surgery, when teens are largely immobile.

"They [adolescents] struggle so hard to achieve independence at home, and it's taken away from them. Being sensitive about that and acknowledging that [is important], because they can't function on their own right after surgery. So they're [depending] on someone to manage their body for them."

Furthermore, health care providers explained that post-surgery, some parents may experience feelings of guilt, distress, anxiety and hyper vigilance, while some teens may experience feelings of depression. Acknowledgement and discussion of these potential outcomes should be covered on the website. *"Just warn families in this website [about what to expect], 'This is normal. It's normal for the parents to hover around their child, but is that necessarily best for that child?"*

Health care providers also suggested including information on typical or common post-operative complications on the website. Clinicians felt that common issues such as constipation, wound infection and pain should be addressed in more detail than other rare or atypical surgical outcomes, such as pulmonary complications, which, they reported, would likely need to be addressed face-to-face with the health care team. While health care providers identified that information regarding both common and rare post-surgical complications is necessary in making an informed decision regarding surgery, they expressed the importance of conveying information in an accurate *and *sensitive manner that did not unduly alarm patients and their families.

Finally, clinicians stressed that website content should be both accurate and realistic, but also sensitive and compassionate in tone. A front line nurse explained, "*My area of expertise is in that initial post-operative period between surgery and home time, so I would just hope that the information is realistic, because what I hear most often is, 'I didn't expect it to be so painful'..., 'they told me there'd be no pain'..., 'I didn't think I'd have to get up this fast'...*".

### Content: Support

Health care providers felt that the website should include a support-based component. Specific sources and means of offering support were identified.

#### Means of Support: Asynchronous text-based message boards and Q & A

Clinicians suggested that support would be best offered via an asynchronous, text-based message board forum. They felt that the message board forum would serve to connect AIS patients with one another, share personal experiences and stories and post frequently asked questions and answers.

"The thing I really liked was the message board idea, so other teens can ask questions or even post questions that have been commonly asked already. So maybe they don't necessarily feel comfortable to pose a question out there, but they can look and view maybe the top 20 asked questions by teens and realize they're not alone for thinking that way. I think that's another really important piece. Because they all feel they are [alone] until we introduce them to somebody else that's going through it."

#### Sources of Support: Peers & Health Care Providers

Clinicians identified the importance of offering professionally-moderated support. They suggested an interactive forum that would provide potential surgical candidates with the opportunity to ask questions of both peers and health care providers. In earlier work in which preferences of adolescents with AIS were also sought, these adolescents similarly requested the inclusion of an interactive support network within the site where they could connect with peers who (i) were considering surgery for AIS, and (ii) had already undergone surgery for AIS [23]. These adolescents felt that many of their informational needs could be met through online social networking such as posted biographies or discussion forums with peers. While health care providers similarly acknowledged the need for an interactive support component, they expressed a greater need for professionally-moderated support than did adolescents. As an example, a health care provider humourously cautioned: *"Yeah, you need a moderator because you know with teenagers...when you're dealing with teenagers and you don't have a moderator...God knows what [will be discussed]."*

### Graphic Design

While health care providers were interested in developing website medical/health *content *targeted at potential scoliosis surgical candidates, they also felt that the website *design *should be developed with the target users in mind. Health care providers felt that this could be achieved through the incorporation of age-appropriate graphics (such as photos, medical illustrations, font styles and colours). Specifically, they expressed that graphics should be heavily integrated with text descriptions and represent the tastes and preferences of the site user.

### Accessibility

The website's content, design and language were noted by health care providers as important aspects to consider in developing a user-friendly and educational online resource. Clinicians supported a website that tailors information and support to potential users while not discriminating based on age, gender, reading level or geographic location. Specifically, the presentation of information and graphics should visually represent and be relevant to both male and female populations, the website should contain content that is applicable to potential scoliosis surgical candidates across Canada, text should be written at a Grade 6 reading level and more advanced information, including research publications should be made available to those who are interested through appropriate links. These health care providers felt that, while the site must be explicitly targeted to the user, it must also provide sufficient latitude or variation of content so as to be useful and accessible to a variety of potential surgical candidates and their families.

## Discussion

Health care providers collectively identified the need for the development of an online information and support resource for adolescents considering surgery for AIS, and their families. Feedback from focus groups identified a well-developed website as a potentially positive and needed adjunct to current clinical care in the aim of supporting patients and their families through the decision-making process as well as surgery and recovery. Specifically, health care providers described four key areas for consideration in the development of this online resource. First, website content, format, layout and graphics should reflect the target user's needs and tastes. Second, the purpose of the website should be clearly identifiable on the home page. Furthermore, the website should have a portal-like navigation feature which allows content to be accessed differentially according to the potential user (adolescent or parent). Third, the website should offer a professionally-moderated interactive support component where the user can access information from both peers and health care providers. Finally, the site should be accessible to a variety of potential surgical candidates and their families, taking into consideration the age, gender, reading level and geographic location of potential users. While the surgical techniques used to correct scoliosis (pedicle screws, hooks, wires etc.) may vary, these variations do not substantially affect the decision-making and information about scoliosis surgery. During website testing, queries from patients and parents will be monitored and technical surgical information will be modified accordingly to address decision-making needs.

While many of the suggestions from health care providers regarding the development of an online resource were congruent with expectations and shared by adolescents with AIS [23], the issue of tailoring website content, format and layout to the target audience was emphasized by health care providers. Specifically, health care providers felt that an online resource has the potential to augment clinical care through expanded access to credible information and support outside of the hospital setting. Yet, they simultaneously recognized that online resources can potentially foster further confusion, frustration or worry among potential patients and their families hindering rather than augmenting clinical care. These undesired outcomes may emerge when online information and support is lacking, contradictory or ill-fitted to the characteristics, needs and contexts of the user. Therefore, a website that is carefully informed by and tailored to the characteristics and needs of the target population across all domains (website content, format, layout and graphics) may best serve to alleviate stress and confusion, reinforce and elaborate on information already received, and offer support. Thus, a website that explicitly responds to the needs and experiences of the surgical candidate and their family through evidence-based, user-centred, participatory design involving all stakeholders, may best serve to empower both patients and families. Furthermore, website content will be reviewed every 3 years and when major new information becomes available to ensure content quality and relevance. Candidates and their families will have enhanced ability to make an informed decision regarding surgery *and *health care providers will gain a resource to complement or augment current clinical practice.

Lastly, similar to adolescent and parent preferences emerging from previous focus groups facilitated in other phases of this project [23], these health care provider focus groups highlighted a strong endorsement for an interactive component comprising peer-based online support. However unlike adolescent patient focus groups, these health care providers strongly recommended professional moderation of the peer support network. This professional facilitation was viewed as an important means for ensuring the credibility of information shared and adherence to guidelines for helpful, safe, productive dialogue.

### Limitations

This study has a few potential limitations. The sample size of the focus groups was relatively small (n = 11) and all participants were from one hospital. However, there was a range in the variety of professionals in the sample, i.e. physicians, nurses, social workers, orthotists, child life specialists; and inpatient and outpatient caregivers were represented. As well, participants brought a wide range and depth of experience in pediatric orthopaedics dealing specifically with the AIS population. The findings between both focus groups were similar, indicating the main issues were identified (i.e. data saturation among these practitioners was achieved). Moreover, careful adherence to established means for ensuring qualitative research trustworthiness increases potential confidence in considering the findings.

## Conclusions

The aim of this study was to develop an online information and support resource for AIS patients considering surgery and their families. Focus group discussion with health care providers revealed not only a unique perspective on the information and support needs of AIS patients and their families but also identified specific content and process-related issues to consider in the development and implementation of the website. Ultimately, consultation with health care providers revealed the importance of an evidence-based, user-centred, participatory design, identifying stakeholder consultation as an essential step towards health-related resource development.

Although web-based health care information, support and interventions certainly do not replace patient-clinician interaction, online health-related resources may well enhance patient-clinician interactions by meeting needs of patients and families that often cannot easily be met during time-limited visits in clinical settings [30]. Accordingly, the integration of well-designed information and support-related material contributes to an online resource that has the potential to enhance knowledge, social support and coping for patients considering surgery for AIS and their families, augment clinical practice and exemplify an innovative patient-centred model for knowledge transfer.

## Competing interests

The authors declare that they have no competing interests.

## Authors' contributions

RM completed data collection, data analysis and drafted the manuscript. JNY and DN reviewed and edited drafts of the manuscript. SD completed data collection and edited drafts of the manuscript. JGW conceived of the study, participated in its design and coordination and reviewed and edited drafts of the manuscript. All authors reviewed and approved the final manuscript.
